# Demography and disorders of the French Bulldog population under primary veterinary care in the UK in 2013

**DOI:** 10.1186/s40575-018-0057-9

**Published:** 2018-05-03

**Authors:** Dan G. O’Neill, Lauren Baral, David B. Church, Dave C. Brodbelt, Rowena M. A. Packer

**Affiliations:** 1 Pathobiology and Population Science, The Royal Veterinary College, Hawkshead Lane, North Mymms, Hatfield, Herts AL9 7TA UK; 2 The Royal Veterinary College, Hawkshead Lane, North Mymms, Hatfield, Herts AL9 7TA UK; 3 Clinical Sciences and Services, The Royal Veterinary College, Hawkshead Lane, North Mymms, Hatfield, Herts AL9 7TA UK

**Keywords:** VetCompass™, Electronic patient record, EPR, Breed, Dog, Epidemiology, Primary-care, Veterinary, Pedigree, Purebred

## Abstract

**Background:**

Despite its Gallic name, the French Bulldog is a breed of both British and French origin that was first recognised by The Kennel Club in 1906. The French Bulldog has demonstrated recent rapid rises in Kennel Club registrations and is now (2017) the second most commonly registered pedigree breed in the UK. However, the breed has been reported to be predisposed to several disorders including ocular, respiratory, neurological and dermatological problems. The VetCompass™ Programme collates de-identified clinical data from primary-care veterinary practices in the UK for epidemiological research. Using VetCompass™ clinical data, this study aimed to characterise the demography and common disorders of the general population of French Bulldogs under veterinary care in the UK.

**Results:**

French Bulldogs comprised 2228 (0.49%) of 445,557 study dogs under veterinary care during 2013. Annual proportional birth rates showed that the proportional ownership of French Bulldog puppies rose steeply from 0.02% of the annual birth cohort attending VetCompass™ practices in 2003 to 1.46% in 2013. The median age of the French Bulldogs overall was 1.3 years (IQR 0.6–2.5, range 0.0–13.0). The most common colours of French Bulldogs were brindle (solid or main) (32.36%) and fawn (solid or main) (29.9%). Of the 2228 French Bulldogs under veterinary care during 2013, 1612 (72.4%) had at least one disorder recorded. The most prevalent fine-level precision disorders recorded were otitis externa (14.0%, 95% CI: 12.6–15.5), diarrhoea (7.5%, 95% CI: 6.4–8.7), conjunctivitis (3.2%, 95% CI: 2.5–4.0), nails overlong (3.1%, 95% CI% 2.4–3.9) and skin fold dermatitis (3.0%, 95% CI% 2.3–3.8). The most prevalent disorder groups were cutaneous (17.9%, 95% CI: 16.3–19.6), enteropathy (16.7%, 95% CI: 15.2–18.3), aural (16.3%, 95% CI: 14.8–17.9), upper respiratory tract (12.7%, 95% CI: 11.3–14.1) and ophthalmological (10.5%, 95% CI: 9.3–11.9).

**Conclusions:**

Ownership of French Bulldogs in the UK is rising steeply. This means that the disorder profiles reported in this study reflect a current young UK population and are likely to shift as this cohort ages. Otitis externa, diarrhoea and conjunctivitis were the most common disorders in French Bulldogs. Identification of health priorities based on VetCompass™ data can support evidence–based reforms to improve health and welfare within the breed.

## Plain English summary

The French Bulldog, despite its name, is a breed of both British and French origin. The breed was first recognised by The Kennel Club in 1906 and has three colourings currently allowed: brindle, fawn and pied. French Bulldogs are currently very popular in the UK and were the second most commonly registered UK pedigree dog breed in 2017. Owners of this breed are attracted to their distinctive appearance, their size being suited to a sedentary lifestyle, and the perception that they are a good companion and children’s breed. However, despite their popularity, the breed has some well-documented health issues, especially in relation to eye, breathing, skin and spinal problems. Collection of health information on large numbers of French Bulldogs attending veterinary practices in the UK would provide reliable data to assist with reforms that aim to improve the health of the breed. The VetCompass™ Programme collects de-identified clinical record data from veterinary practices in the UK for research to improve animal welfare.

French Bulldogs made up 2228 (0.49%) of 445,557 study dogs under veterinary care during 2013. By calculating the French Bulldog proportion of dogs born each year that attended VetCompass™ practices, the study showed that French Bulldogs accounted for just 0.02% of puppies born in 2003 but rose to comprise 1.46% of all puppies born in 2013. The most common colours of the study French Bulldogs were brindle (solid or main) (32.36%) and fawn (solid or main) (29.9%). Of the 2228 French Bulldogs under veterinary care during 2013, 1612 (72.4%) had at least one disorder recorded. The most common disorders recorded were ear infections (14.0%), diarrhoea (7.5%) and conjunctivitis (3.2%). Skin problems were the most commonly reported group of disorders (17.9%). This study of over two thousand French Bulldogs provides a framework to identify the most important health priorities in French Bulldogs in the UK and can assist with reforms to improve health and welfare within the breed.

## Background

The French Bulldog, despite its gallic name is a breed of both British and French origin, with ancestral roots from the Toy Bulldog of Britain in the 1850s [1]. Following the introduction of the Toy Bulldog to Northern France during the Industrial Revolution, crosses with other brachycephalic (short-muzzled) breeds are thought to have occurred, resulting in the French Bulldog breed as known today. The French Bulldog returned to Britain at the end of the nineteenth century and was first formally recognised by The Kennel Club (KC) in 1906 with three colourings currently allowed: brindle, fawn and pied [1].

The French Bulldog was the UK’s second most commonly registered KC breed for 2017, exceeded only by the Labrador Retriever in popularity. This followed a dramatic rise to popularity, with annual registration data from the UK Kennel Club highlighting an over thirty-fold increase in registrations over the past decade, from just 692 registrations in 2007 to 21,470 registrations in 2016 [2]. Current popularity trajectories suggest that the French Bulldog will become the most registered breed in the UK by the end of 2018, stealing a title that the Labrador Retriever has held since 1990. It is worth noting that a rising breed popularity with a consequently young population means that current disorder profiles will reflect the diseases of youth and are likely to shift more towards diseases of aging as popularity increases decline. Although national dog populations often remain largely stable over long periods, individual breeds can show substantial, and often culturally driven, surges and decreases in popularity in sort periods (Herzog et al. 2004). Social influence (fashion) has a strong effect on the popularity of dog breeds, and is often related to media exposure, e.g. featuring in movies [3, 4]. Indeed, celebrity ownership of French Bulldogs and subsequent media exposure is often anecdotally cited as a driving force behind their rise. The distinctive appearance of this brachycephalic breed has been reported to be a key factor influencing their popularity [5, 6]; but despite their appearance-driven popularity, the French Bulldog is reportedly affected by a number of health problems associated with their conformation, including their short muzzles and wide, prominent eyes [7, 8].

French Bulldogs are reported as predisposed to health disorders including brachycephalic obstructive airway syndrome (BOAS) [7, 9–11], dystocia [12], corneal ulceration [8, 13], patellar luxation [14] and a range of spinal diseases including type I intervertebral disk herniation [15], spinal disease associated with vertebral malformations [16, 17] and spinal arachnoid diverticulum [15]. It is worth noting that many of these reported predispositions increase in prevalence as dogs age and therefore analyses of younger populations may artifactually appear to show reduced prevalence compared with more mature populations. Indeed, French Bulldogs have been reported with at least ten breed predispositions to disease [18]. In recognition of these breed health challenges, the UK KC originally listed the French Bulldog as a category 3 breed (the highest category) in its ‘Breed Watch’ system which aims to identify points of concern for individual breeds ‘where some dogs have visible conditions or exaggerations that can cause pain or discomfort’. Since 2013, the French Bulldog has been moved to category 2; however, a range of points of concern remain that are highlighted for special attention by show judges including ‘dogs showing respiratory distress including difficulty in breathing or labored breathing, exaggerated roach in the top line, excessively prominent eyes, hair loss or scarring from previous dermatitis, incomplete blink, incorrect bite, inverted tail, lack of tail, overly short neck, pinched nostrils, screw tail, signs of dermatitis in skin folds and tight tail’ [1].

Using veterinary clinical data from the VetCompass™ Programme [19], this study aimed to characterise the demography, longevity and common disorders of the general population of French Bulldogs under veterinary care in the UK, with an exploratory comparative focus on differences between males and females. The results from the current study could provide a reliable framework to assist reforms in breeding practices and ultimately contribute to improved health and welfare of French Bulldogs.

## Methods

The study population included all dogs under primary veterinary care at clinics participating in the VetCompass™ Programme during 2013. Dogs under veterinary care were defined as those with either a) at least one electronic patient record (EPR) (VeNom diagnosis term, free-text clinical note, treatment or bodyweight) recorded during 2013 or b) at least one EPR recorded both before and after 2013. The VetCompass™ Programme collates de-identified EPR data from primary-care veterinary practices in the UK for epidemiological research [19]. Collaborating practices can record summary diagnosis terms during episodes of care from an embedded VeNom Code list [20]. Data fields available to VetCompass™ researchers for each dog include a unique animal identifier along with species, breed, date of birth, colour, sex, neuter status and bodyweight, and clinical information from free-form text clinical notes, summary diagnosis terms (VeNom codes) and treatment with relevant dates.

A prevalence study design derived from the cohort clinical data of dogs registered at participating practices was used to estimate the one-year period prevalence of the most commonly diagnosed disorders [21]. Sample size calculations estimated that 2136 dogs would be needed to detect a sex effect for a disorder with 2.5% expected prevalence in the protected sex at a 95% confidence level with 80% power assuming a 1:1 ratio of males to females [22]. Ethics approval was obtained from the Royal Veterinary College Clinical Research Ethical Review Board (CRERB) (reference number 2015/1369).

Dogs recorded as French Bulldog breed were categorised as French Bulldog and all remaining dogs were categorised as non-French Bulldog. *All-age Bodyweight* (Kg) described recorded all available bodyweight and date combinations. *Adult Bodyweight* (Kg) described the mean bodyweight recorded from all bodyweight data for dogs aged over 18 months and was categorised into 6 groups (< 9.0, 9.0 to < 11.0, 11.0 to < 13.0, 13.0 to < 15.0, 15.0 to < 17.0, ≥ 17.0). N*euter* described the status of the dog (entire or neutered) at the final EPR. *Age* described the age at the final date under veterinary care during 2013 (December 31st, 2013) and was categorised alternatively into 7 groups (< 1.0, 1.0 to < 2.0, 2.0 to < 3.0, 3.0 to < 6.0, 6.0 to < 9.0, 9.0 to < 12.0, ≥ 12.0) and also into a binary age categorization (< 2.0 and ≥ 2.0).

The list of unique French Bulldog animal identification numbers was randomly ordered and the clinical records of all animals were reviewed manually in detail to extract the most definitive diagnoses recorded for all disorders that existed during 2013 [10]. Elective (e.g. neutering) or prophylactic (e.g. vaccination) clinical events were not included. No distinction was made between pre-existing and incident disorder presentations. Disorders described within the clinical notes using presenting sign terms (e.g. ‘vomiting’ or ‘vomiting and diarrhoea’), but without a formal clinical diagnostic term being recorded, were included using the first sign listed (e.g. vomiting). Mortality data (recorded cause, date and method of death) were extracted on all deaths at any date during the available EPR data.

The extracted diagnosis terms were mapped to a dual hierarchy of diagnostic precision for analysis: fine-level precision and grouped-level precision as previously described [10]. Briefly, fine-level precision terms described the original extracted terms at the maximal diagnostic precision recorded within the clinical notes (e.g. *inflammatory bowel disease* would remain as *inflammatory bowel disease*). Grouped-level precision terms mapped the original diagnosis terms to a general level of diagnostic precision (e.g. *inflammatory bowel disease* would map to *gastro-intestinal*).

Following data checking for internal validity and cleaning in Excel (Microsoft Office Excel 2013, Microsoft Corp.), analyses were conducted using Stata Version 13 (Stata Corporation). The sex, neuter status, age, colour and adult bodyweight for French Bulldogs under veterinary care during 2013 were described. Annual proportional birth rates described the relative proportion of French Bulldogs compared with all dogs that were born in each year from 2003 to 2013 from the cohort that were under veterinary care in 2013. All-age bodyweight data with their associated dates were used to generate individual bodyweight growth curves for male and female French Bulldogs by plotting age-specific bodyweights and were overlaid with a cross medians line plot using the Stata *mband* command.

One-year (2013) period prevalence values were reported along with 95% confidence intervals (CI) that described the probability of diagnosis at least once during 2013. The CI estimates were derived from standard errors based on approximation to the normal distribution for disorders with ten or more events [23] or the Wilson approximation method for disorders with fewer than ten events [24]. Prevalence values were reported overall, separately for males and females and also separately for < 2.0 years and ≥ 2.0 years. The chi-square test was used to compare categorical variables and the Students t-test or Mann-Whitney U test to compare continuous variables as appropriate [23]. Statistical significance was set at the 5% level.

## Results

### Demography and mortality

**Fig. 1:**
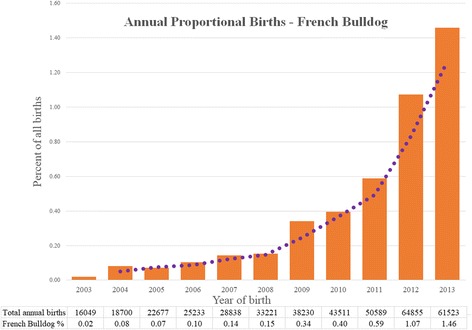
Annual proportional birth rates (2003–2013) for French Bulldogs (*n* = 2228) among all dogs (*n* = 455,557) attending UK primary-care veterinary clinics participating in the VetCompass™ Programme. A moving average trendline calculated from 2 periods is overlaid

**Fig. 2:**
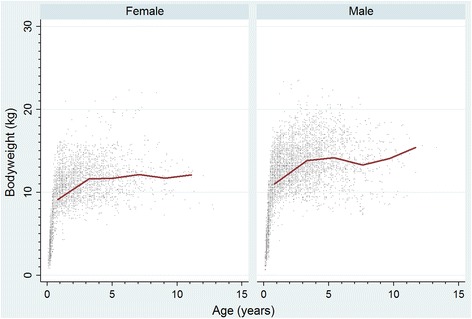
Bodyweight growth curves overlaid with a cross medians line plot for female and male French Bulldogs attending UK primary-care veterinary clinics participating in the VetCompass™ Programme. (Weight values: Females *n* = 3413, Males *n* = 5276)

The study population of 455,557 dogs from 304 clinics in the VetCompass™ database under veterinary care during 2013 included 2228 (0.49%) French Bulldogs. Of these French Bulldogs with information available, 1047 (48.5%) were female and 461 (26.2%) were neutered. Males were more likely to be neutered than females (30.7% versus 21.8%, *P* <  0.001). The mean adult bodyweight overall was 12.7 kg (standard deviation [SD] 2.5 kg). The mean adult bodyweight of males (13.7 kg, SD 2.4 kg) was heavier than for females (11.5 kg, SD 2.1 kg) (*P* <  0.001). The median age of the French Bulldogs overall was 1.3 years (IQR 0.6–2.5, range 0.0–13.0). The most common recorded colours were brindle (solid or main) (*n* = 671, 32.36%) and fawn (solid or main) (621, 29.9%) (Table 1). Data completeness varied across the variables assessed: age 98.71%, sex 99.5%, neuter 79.0%, colour 93.4% and all-age bodyweight 66.1%. Annual proportional birth rates showed that French Bulldogs increased steeply from 0.02% of the annual VetCompass™ birth cohort in 2003 to 1.46% in 2013 (Fig. 1). The median bodyweight across all ages was higher for males (12.3 kg, IQR: 9.5–14.3, range: 0.7–23.5) than for females (10.3 kg, IQR: 8.0–12.0, range: 0.8–22.3) (*P* <  0.001). Bodyweight growth curves based on 3413 bodyweight values from 684 females and 5276 bodyweight values from 783 males showed that French Bulldog puppies grow rapidly during their first year but continue to gain further weight up to three years (Fig. 2).

**Table 1:** Demography of French Bulldogs under primary veterinary care at practices participating in the VetCompass™ Programme in the UK from January 1st, 2013 to December 31st, 2013 (*n* = 2228)

Variable	Category	Count^a^	Percent
Sex	Female	1074	48.5
	Male	1142	51.5
Female neuter	Entire	671	78.2
	Neutered	187	21.8
Male neuter	Entire	618	69.3
	Neutered	274	30.7
Female adult bodyweight (aged ≥18 months) (kg)	< 9.0	32	7.8
	9.0 to < 11.0	138	33.7
	11.0 to < 13.0	154	37.7
	13.0 to < 15.0	63	15.4
	15.0 to < 17.0	18	4.4
	≥ 17.0	4	1.0
Male adult bodyweight (aged ≥18 months) (kg)	< 9.0	4	0.8
	9.0 to < 11.0	51	10.0
	11.0 to < 13.0	156	30.7
	13.0 to < 15.0	154	30.3
	15.0 to < 17.0	86	16.9
	≥ 17.0	58	11.4
Age (years)	< 1.0	840	38.2
	1.0 to < 2.0	646	29.4
	2.0 to < 3.0	276	12.6
	3.0 to < 6.0	328	14.9
	6.0 to < 9.0	81	3.7
	9.0 to < 12.0	25	1.1
	≥ 12.0	4	0.2
Colour	Brindle- solid or main	671	32.3
	Fawn - solid or main	621	29.9
	Black - solid or main	383	18.4
	Pied	256	12.3
	Blue - solid or main	86	4.1
	Liver/chocolate - solid or main	63	3.0

^a^Count covers dogs with available data

There were 98 deaths recorded during the study of which 92 had a recorded sex and age at death. Longevity and mortality described the distribution of ages and causes of death within the current study of a young and emerging population of French Bulldogs and reflect this specific young population. The longevity and mortality profiles of a stable population are likely to be quite different and to reflect substantially greater longevity with a shift towards mortality from diseases of aging. The median longevity of French Bulldogs overall was 3.6 years (IQR 1.8–6.1, range 0.0–12.2). The median longevity of females (4.0 years, IQR 2.1–6.1, range 0.0–12.2, *n* = 38) did not differ to males (3.5 years, IQR 1.6–6.1, range 0.0–11.5, *n* = 54) (*P* = 0.918). There were 14 (14.3%) deaths that did not have a cause of death stated. Of the remaining 84 deaths, the most common causes of death described at a grouped-precision level were brain disorder (*n* = 10, prevalence 11.9%) and spinal cord disorder (8, 9.5%) (Table 2).

**Table 2:** Causes of mortality in French Bulldogs with a recorded cause of death under primary-care veterinary at UK practices participating in the VetCompass™ Programme from January 1st, 2013 to December 31st, 2013 (*n* = 84)

Grouped-level disorder	Count	Percent	Median age (years) at death
Brain disorder	10	11.9	2.1
Spinal cord disorder	8	9.5	4.0
Lower respiratory tract disorder	6	7.1	0.9
Mass lesion	6	7.1	7.0
Upper respiratory tract (URT) disorder	6	7.1	2.5
Undesirable behaviour disorder	5	6.0	2.1
Traumatic injury	5	6.0	2.6
Vertebral spinal disorder	5	6.0	5.3
Complication associated with clinical care procedure	4	4.8	2.4
Enteropathy	4	4.8	0.8
Other	25	29.8	
Total	84	100.0	3.6

The median age of the overall French Bulldog population was 1.3 years

### Disorder prevalence

The EPRs of all 2228 French Bulldogs were manually examined to extract all recorded disorder data for 2013. There were 1612 (72.4%) French Bulldogs with at least one disorder recorded during 2013 while the remaining 27.6% had no disorder recorded and either presented for prophylactic management only or did not present at all during 2013. The median annual disorder count per French Bulldog during 2013 was 1 disorder (IQR 0–2, range 0–12). There was some evidence of higher annual disorder count in males (median 1, IQR 0–2, range 0–11) than females (median 1, IQR 0–2, range 0–12) (*P* = 0.022).

The study included 3380 unique disorder events recorded during 2013 that encompassed 343 distinct fine-level disorder terms. The most prevalent fine-level precision disorders recorded were otitis externa (*n* = 312, prevalence 14.0%, 95% CI: 12.6–15.5), diarrhoea (167, 7.5%, 95% CI: 6.4–8.7), conjunctivitis (71, 3.2%, 95% CI: 2.5–4.0), nails overlong (69, 3.1%, 95% CI% 2.4–3.9) and skin fold dermatitis (66, 3.0%, 95% CI% 2.3–3.8). Males had a higher probability of diagnosis than females for 8 of the 26 most common fine-level precision disorders (conjunctivitis, prolapsed nictitans gland, BOAS, aggression, vomiting, upper respiratory tract (URT) disorder, claw injury and stenotic nares) while females did not have higher prevalence than males for any fine-level precision disorders (Table 3). Age was significantly associated with the prevalence of 10/26 (38.5%) the most common fine-level precision disorders. The direction of association was split equally across these 10 discordant disorders with 5 disorders having higher prevalence in dogs aged < 2 years (diarrhoea, prolapsed nictitans gland, infectious canine tracheobronchitis, ear discharge, demodicosis) and 5 disorders having higher prevalence in dogs aged ≥2 years (otitis externa, skin fold dermatitis pododermatitis, ulcerative keratitis, atopic dermatitis) (Table 4).

**Table 3:** Prevalence of the most common disorders at a *fine-level of diagnostic precision* recorded in French Bulldogs (*n* = 2228) attending UK primary-care veterinary practices participating in the VetCompass™ Programme from January 1st, 2013 to December 31st, 2013

Fine-level disorder	Count	Overall prevalence %	95% CI^a^	Female prevalence %	Male prevalence %	*P*-value
Otitis externa	312	14.0	12.6–15.5	13.7	14.4	0.648
Diarrhoea	167	7.5	6.4–8.7	7.0	8.1	0.339
Conjunctivitis	71	3.2	2.5–4.0	2.4	3.9	0.042
Nails overlong	69	3.1	2.4–3.9	2.6	3.6	0.183
Skin fold dermatitis	66	3.0	2.3–3.8	2.6	3.3	0.319
Anal sac impaction	64	2.9	2.2–3.7	3.1	2.7	0.615
Upper respiratory tract (URT) infection	61	2.7	2.1–3.5	2.1	3.3	0.088
Pyoderma	60	2.7	2.1–3.5	1.9	3.5	0.017
Prolapsed nictitans gland	57	2.6	1.9–3.3	2.4	2.7	0.663
Pododermatitis	55	2.5	1.9–3.2	2.2	2.7	0.468
Brachycephalic obstructive airway syndrome (BOAS)	54	2.4	1.8–3.2	1.4	3.4	0.002
Colitis	53	2.4	1.8–3.1	2.1	2.6	0.455
Aggression	51	2.3	1.7–3.0	0.8	3.7	< 0.001
Heart murmur	49	2.2	1.6–2.9	1.7	2.7	0.097
Vomiting	48	2.2	1.6–2.8	1.4	2.9	0.016
Infectious canine tracheobronchitis	47	2.1	1.6–2.8	2.2	2.0	0.719
Upper respiratory tract (URT) disorder	47	2.1	1.6–2.8	1.4	2.7	0.030
Patellar luxation	46	2.1	1.5–2.7	1.9	2.3	0.494
Ulcerative keratitis	46	2.1	1.5–2.7	1.7	2.5	0.200
Claw injury	44	2.0	1.4–2.6	1.3	2.6	0.026
Atopic dermatitis	44	2.0	1.4–2.6	1.7	2.3	0.311
Gastroenteritis	43	1.9	1.4–2.6	2.0	1.9	0.961
Ear discharge	42	1.9	1.4–2.5	1.6	2.2	0.296
Alopecia	41	1.8	1.3–2.5	1.9	1.8	0.968
Demodicosis	37	1.7	1.2–2.3	1.5	1.8	0.522
Stenotic nares	37	1.7	1.2–2.3	0.9	2.4	0.009

The *P*-value reflects prevalence comparison between females and males

^a^CI confidence interval

**Table 4:** Comparison of the prevalence of the most common disorders at a *fine-level of diagnostic precision* between French Bulldogs aged under 2 years (*n* = 1486) and aged at/above 2 years (*n* = 714) attending UK primary-care veterinary practices participating in the VetCompass™ Programme from January 1st, 2013 to December 31st, 2013

Fine-level disorder	Median age (years) of affected dogs	Count (%) at < 2 years	Count (%) at ≥2 years	*P*-value
Otitis externa	1.75	172 (11.6)	140 (19.6)	< 0.001
Diarrhoea	0.95	142 (9.6)	24 (3.4)	< 0.001
Conjunctivitis	1.36	50 (3.4)	21 (2.9)	0.599
Nails overlong	1.33	46 (3.1)	23 (3.2)	0.874
Skin fold dermatitis	1.88	36 (2.4)	30 (4.2)	0.022
Anal sac impaction	1.81	36 (2.4)	28 (3.9)	0.050
Upper respiratory tract (URT) infection	1.71	28 (1.9)	19 (2.7)	0.238
Pyoderma	1.38	43 (2.9)	17 (2.4)	0.489
Prolapsed nictitans gland	0.95	50 (3.4)	6 (0.9)	< 0.001
Pododermatitis	2.10	26 (1.8)	29 (4.1)	0.001
Brachycephalic obstructive airway syndrome (BOAS)	1.67	32 (2.2)	21 (2.9)	0.259
Colitis	1.05	45 (3.0)	8 (1.1)	0.006
Aggression	2.21	23 (1.6)	27 (3.8)	0.001
Heart murmur	0.81	37 (2.5)	11 (1.5)	0.154
Vomiting	1.34	35 (2.4)	13 (1.8)	0.422
Infectious canine tracheobronchitis	1.09	38 (2.6)	8 (1.1)	0.027
Upper respiratory tract (URT) disorder	1.71	28 (1.9)	19 (2.7)	0.238
Patellar luxation	1.66	26 (1.8)	19 (2.7)	0.157
Ulcerative keratitis	2.79	19 (1.3)	27 (3.8)	< 0.001
Claw injury	1.76	24 (1.6)	20 (2.8)	0.063
Atopic dermatitis	3.08	16 (1.1)	28 (3.9)	< 0.001
Gastroenteritis	1.19	27 (1.8)	16 (2.2)	0.501
Ear discharge	1,12	37 (2.5)	4 (0.6)	0.002
Alopecia	1.45	27 (1.8)	13 (1.8)	0.995
Demodicosis	0.91	33 (2.2)	4 (0.6)	0.005
Stenotic nares	1.84	21 (1.4)	16 (2.2)	0.157

The median age of the overall French Bulldog population was 1.3 years

There were 59 distinct grouped-level precision disorder terms recorded. The most prevalent grouped-level precision disorders were cutaneous (*n* = 399, prevalence: 17.9%, 95% CI: 16.3–19.6), enteropathy (372, 16.7%, 95% CI: 15.2–18.3), aural (364, 16.3%, 95% CI: 14.8–17.9), URT (282, 12.7%, 95% CI: 11.3–14.1) and ophthalmological (234, 10.5%, 95% CI: 9.3–11.9). Males were more likely than females to be diagnosed with 6 of the 15 most common grouped-level precision disorders (cutaneous, enteropathy, URT, nail disorder, undesirable behavior and lower respiratory tract) while females did not have higher prevalence than males for any grouped-level precision disorders (Table 5).

**Table 5:** Prevalence of the most common *grouped-level disorders* recorded in French Bulldogs (*n* = 2228) attending UK primary-care veterinary practices participating in the VetCompass™ Programme from January 1st, 2013 to December 31st, 2013

Grouped-level disorder	Count	Overall prevalence	95% CI^a^	Female prevalence %	Male prevalence %	*P*-value
Cutaneous	399	17.9	16.3–19.6	16.0	19.9	0.018
Enteropathy	372	16.7	15.2–18.3	14.5	18.9	0.006
Aural	364	16.3	14.8–17.9	16.0	16.7	0.652
Upper respiratory tract (URT)	282	12.7	11.3–14.1	10.0	15.2	< 0.001
Ophthalmological	234	10.5	9.3–11.9	9.3	11.7	0.073
Nail disorder	119	5.3	4.4–6.4	4.2	6.5	0.017
Musculoskeletal	105	4.7	3.9–5.7	4.2	5.3	0.239
Parasite infestation	98	4.4	3.6–5.3	4.2	4.6	0.676
Anal sac disorder	85	3.8	3.1–4.7	3.9	3.8	0.859
Undesirable behaviour	74	3.3	2.6–4.2	1.5	5.1	< 0.001
Traumatic injury	68	3.1	2.4–3.9	2.5	3.6	0.142
Lower respiratory tract	53	2.4	1.8–3.1	1.7	3.0	0.043
Cardiac	52	2.3	1.7–3.0	1.8	2.9	0.082
Mass lesion	45	2.0	1.5–2.7	2.5	1.5	0.084
Dental	43	1.9	1.4–2.6	2.0	1.9	0.961

The *P*-value reflects prevalence comparison between females and males

^a^CI confidence interval

## Discussion

This study of over two thousand animals is the largest analysis of breed health in French Bulldogs based on primary-care veterinary records to date. The results highlight steeply rising French Bulldog ownership in the UK, with French Bulldogs comprising over 1.46% of all dogs born in 2013 attending veterinary practices, although not all of these may have been born in the UK. These findings are consistent with registration data from The Kennel Club that highlights the French Bulldog currently as the most rapidly rising pedigree breed in the UK, surpassing other popular small brachycephalic breeds such as the Pug. This trend has also been observed internationally, with shorter, smaller and flatter faced dog breeds also becoming more popular in Australia between 1986 and 2013 [25].

Both in the United States of America (USA) and the UK, breed popularity appears to lack direct associations with functional traits (e.g. health, trainability) [3, 26] whilst displaying a concerning tendency for more popular breeds to have greater numbers of inherited disorders [26]. For owners of small brachycephalic breeds including the French Bulldog, the breeds’ appearance, size being suited to the owners’ lifestyle and behavioural traits (good dog breed for children and good companion breed) have been reported as more highly influential to breed choice by owners than either health or longevity [5]. In addition, French Bulldog owners may represent examples of ‘extrinsically motivated’ owners, who acquire dogs as a means of obtaining status and attention from other people, due to the distinctiveness or ‘cuteness’ of the dog, and may often perceive their dog as helpless and in need of care and control [6]. With several of the most common disorders in French Bulldogs linked to their physical conformation (e.g. URT disease and ophthalmological conditions), the increasing popularity of this breed is not necessarily a benign phenomenon. Increased demand for dogs with extreme conformational features is suggested to be detrimental to these dogs’ welfare both because of directly linked disorder risk and also because steeply increasing demand may contribute to suboptimal breeding and welfare standards as breeders and suppliers rapidly attempt to fulfil the heightened consumer demand [27]. With dogs born in high-volume commercial breeding establishments experiencing an increased incidence of behavioural and emotional problems that cause distress in adulthood compared with dogs from other sources, dramatic increases in popularity are a welfare concern for any breed [28]. Consequently, surveillance of the health of the general population of French Bulldogs in the UK is of increasing importance to provide evidence on both breed and husbandry related welfare concerns.

The most common disorders identified in the current study for French Bulldogs were otitis externa, diarrhoea, conjunctivitis, overlong nails and skin fold dermatitis. These results provide a framework to identify health priorities from a prevalence perspective in French Bulldogs that can contribute positively to reforms that aim to improve health and welfare within the breed. Although longevity did not differ between males and females, marked sex differences in disorder prevalence were observed, with males were more likely than females to be diagnosed 8 of the 26 most common fine-level precision disorders, and 6 of the 15 most common grouped-level precision disorders. These additional sex-based prevalence data can highlight those disorders that would benefit from special focus within specific sexes in order to contribute to improved French Bulldog health and welfare as well as assisting decision-making by veterinarians and owners on the most appropriate sex selection [10]. The current study data do not provide a strong rationale to explain the disorder prevalence differences between males and females but these may be associated with differing body sizes (13.7 kg in males versus 11.5 kg in females) or hormonal profiles between the sexes [29]. Sex-associated differences in dogs for disorder prevalence in multi-disorder studies have also been reported for Rottweilers [30], Border Terriers [31] and German Shepherd Dogs [32] and may represent an under-explored area of research that could enhance our understanding of disease causality.

URT disorders were the fourth most common grouped-level disorder, reported in 12.7% of French Bulldogs. The most commonly recorded fine level disorders within the URT group included brachycephalic obstructive airway syndrome (BOAS) (2.4%), URT disorder (2.1%), and stenotic nares (1.7%). BOAS encompasses a range of primary or secondary disorders that may include stenotic nares, enlarged tonsils, elongated soft palate, everted lateral saccules of the larynx, narrowed rima glottides, collapse of the larynx and tracheal hypoplasia [33, 34]. BOAS is considered a major animal welfare concern, with the lives of affected animals negatively impacted both while awake and asleep by clinical signs including chronic breathlessness, exercise intolerance, eating difficulties and disrupted sleeping including periods of apnoea [35]. The relatively low prevalence of recorded diagnoses of BOAS and other respiratory problems in the current primary-care population using a retrospective observational study design is in sharp contrast with the findings from some other prospective clinical studies. A prospective study of BOAS in the UK reported that 70% of French Bulldogs attending a referral veterinary hospital and 75% of a general population of French Bulldogs had BOAS based on clinical history, owner questionnaire and clinical examination [7]. A UK clinical study using whole-body barometric plethysmography reported that 89.9% of French Bulldogs tested were affected by BOAS to some extent, with 53.9% exhibiting clinically relevant disease [9]. These latter data suggest that many truly BOAS-affected French Bulldogs may be accepted as ‘normal for breed’ by owner and the veterinary profession because the pervasively high true prevalence of the disorder may conflate perceptions of ‘typically expected’ and ‘desirably expected’. The authors would strongly encourage veterinarians, breeders and owners to avoid the use of the word ‘normal’ with its inference of acceptability in relation to breed-related health features and move instead to alternative terms such as ‘typical’ or ‘commonplace’. Indeed, only 42% of owners of dogs affected by BOAS perceive that their dog has a breathing problem; as such, it is possible that only the most severely affected cases may receive a formal BOAS diagnosis in the primary-care setting [36]. Psychological desensitisation in veterinarians to URT in brachycephalic dogs may result from chronic exposure to common clinical signs of BOAS (e.g. increased and/or abnormal respiratory noise) in brachycephalic breeds such as the French Bulldog that potentially leads to underreporting in clinical notes. Unfortunately, this may also contribute to the sub-optimal clinical management of individual affected dogs if the condition is under-recognised and not discussed with clients. An earlier VetCompass™ study reported that 20% of French Bulldogs had least one URT disorder recorded over a 4.5 year study period, compared to the one year period of the current study, so it is possible that more French Bulldogs in the current population would go on to be diagnosed with URT disorders with a longer study period [34]. The current study reports that older dogs are significantly more likely to have a diagnosis of BOAs than younger dogs and suggests that the UK population of French Bulldogs are likely to show substantially higher levels of BOAS as the current cohort of dogs ages.

Dermatological disorders were a commonly recorded grouped-level event in French Bulldogs, with 17.9% of the study population affected. At the fine-level of disorder reporting, skin fold dermatitis was the fifth most common disorder (3.0%), followed by pyoderma (2.7%), pododermatitis (2.5%) and atopic dermatitis (2.0%). Skin disease is well-recognised as a breed-specific issue in French Bulldogs, with both hair loss or scarring from previous dermatitis and signs of dermatitis in skin folds listed as points of concern in ‘Breed Watch’ for the breed [1]. Skin fold dermatitis may occur at any site on the body where excessive skin wrinkling causes skin-on-skin contact, including the facial region of brachycephalic dogs or in skin folds around absent, short or screw-tails [37]. Skin fold dermatitis (intertrigo) was also a common finding in a previous VetCompass™ study of Pugs [38], another brachycephalic breed where skin folds are common, particularly on the face (referred to as ‘over nose wrinkle’) [1]. Although an over nose wrinkle is not explicitly encouraged, The Kennel Club (UK) French Bulldog breed standard specifies that skin on the skull and forehead “should be supple enough to allow fine wrinkling” [1]; and the American Kennel Club breed standard describes “heavy wrinkles forming a soft roll over the extremely short nose” [39]. In addition to conformation-related skin disorders, the French Bulldog has also been reported as both predisposed to canine atopic dermatitis [40] and also reported as developing clinical signs of atopic dermatitis earlier than other breeds, which may suggest a higher genetic predisposition [41]. The median age of dogs affected with atopic dermatitis in the current study was 3.8 years and the condition was significantly more likely in dogs older than 2 years (Table 4). In a breed such as the French Bulldog where immune-mediated skin disease is common, ensuring their conformation does not exacerbate existing skin disorders, or directly cause skin disease in itself should be of priority for breed welfare. As proposed in the Independent Inquiry into Dog Breeding, “*Where a problem within a breed already exists, the Breed Standard should be amended specifically to encourage the selection for morphologies that will improve the welfare status of the breed*” [42]. Amending breed standards and breeding away from skin folds may go some way to encourage healthier skin conformation and improve breed health.

Ophthalmological disorders (10.5%) were the fifth most common grouped-level disorder for French Bulldogs, with conjunctivitis (3.2%), prolapsed nictitans gland (2.6%) and ulcerative keratitis (2.1%) in the top 20 most common fine-level disorders for the breed. A previous VetCompass™ study of corneal ulceration reported that French Bulldogs had the 8th highest breed prevalence for this disorder (prevalence: 1.87%) and had a five-times increased odds of diagnosis with corneal ulceration compared with crossbreeds [13]. The median age at diagnosis of corneal ulceration across all breeds in the UK has been reported as 4.9 years [13]. This value is higher than the median of 2.79 years reported in the current study and may reflect the relative youth of the French Bulldog population. A prospective clinical study reported that 15.4% of French Bulldogs were affected by corneal ulcers [8], with conformational risk factors for corneal ulcers across dog breeds also identified in the same study. These included the presence of a nasal fold, brachycephalic skull shape and wide eyelid openings, all of which are commonly observed in French Bulldogs [8]. The UK Kennel Club has taken efforts to redress these associations, with ‘excessively prominent eyes’ and ‘incomplete blink’ (lagophthalmos) identified as points of concern for the French Bulldog in Breed Watch [1]. Given that the canine cornea is densely innervated by nociceptive afferent axons and that in a previous study, 69.1% of canine corneal ulcers cases had either pain recorded in their notes and/or received pain management [13], ensuring that skull, eyelid and eye conformation do not predispose dogs to these welfare-relevant disorders is of major welfare importance.

Aggression was the thirteenth most common fine-level disorder recorded in French Bulldogs (2.3%). In contrast, aggression did not feature among the 25 most common fine-level disorders in Pugs in the UK [38]. The differing age distributions of the two study populations (3.0 years median age of Pug population versus 1.3 years median age in the current French Bulldog study) are unlikely to explain the differing aggression prevalence since the current study identified that aggression was more common in dogs older than 2 years compared with dogs younger than 2 years [43]. In addition, both studies had similar ratios of males and females overall. This relatively high level of aggression in the French Bulldog is somewhat surprising in light of the finding that owners of French Bulldogs were influenced to buy their breed by a perception that they were a ‘good companion breed’ and ‘good with children’ [5], with their temperament described as a ‘deeply affectionate’ in their UK breed standard [1]. As such, further data on the behaviour of French Bulldogs are required to more precisely characterise the types of aggression seen and explore the underlying motivation for these behaviours. As in previous studies, a higher male prevalence of aggression was observed in French Bulldogs (males 3.7% vs. females 0.8%), which may be related to androgens promoting competitive behaviour [44, 45].

During the one-year period of surveillance (2013) of this study, 27.6% of French Bulldogs under veterinary care did not have any disorders recorded and were instead either presented for routine or prophylactic veterinary care or did not attend the veterinary clinic at all. This value is comparable with the 24% of dogs without any recorded disorders that was reported across a random sample of all breeds in the VetCompass™ database [46]. With well-documented health concerns in French Bulldogs, it is perhaps surprising that the proportion of French Bulldogs with at least one disorder recorded is not higher than for the overall population. However, this may be explained by the younger age of French Bulldogs in the current study (median age: 1.3 years) compared with the overall dog population in the previous study (median age: 4.5 years). It has previously been documented that French bulldogs are very young when they exhibit their first veterinary care event (less than two years compared to around five years for all breeds [6]), which may reflect that the health problems of the breed are more related to congenital disorders instead of acquired and age-related diseases [6]. The young median age of 1.3 years in the current study may also explain why periodontal disease, the second most common disorder affecting dogs overall in the UK [46] did not feature amongst the common disorders of French Bulldogs. In addition, although spinal cord disorders were a common cause of death in the current study, and Hansen type I disc herniation (IVDH) has been reported to be the most common neurological disease in French Bulldogs [15], spinal disorders did not feature amongst the most common disorders in the present study. This is again likely due to the young age of this study population, with 81% of French Bulldogs affected by IVDH aged 3 years old or more [15]. The current study identified that 38% of the common disorders recorded in French Bulldogs were associated with age with equal proportions showing higher prevalence in older and younger dogs. It is planned to repeat the current study in the future and this may identify shifting disorder profiles towards diseases of aging and away from disorders of youth assuming that breed popularity wanes over time.

The study had some limitations that have been previously reported [46]. The study was unable to segregate and compare between Kennel Club registered and unregistered dogs but ongoing efforts underway within the VetCompass™ Programme should enable such distinctions in the future and could contribute to greater clarity on the health comparisons between these two groups. Studies based on reviews of medical records of animals may under-estimate the true disease burden by predominantly including those more severely affected animals that warrant veterinary management and there may be reduced reporting of less severely affected animals that may be less likely to be clinically presented [47]. Clinical variants of some diseases may be recorded using distinct disorder terms and therefore the overall prevalence for these diseases may be fragmented into separate prevalence values for each of multiple more-specific diagnostic term, giving the illusion of lower prevalence [10].

## Conclusion

This study of over two thousand French Bulldogs documented steeply rising ownership of French Bulldogs in the UK and provides important disorder information on the general population of French Bulldogs. The most common disorders in French Bulldogs were otitis externa, diarrhoea and conjunctivitis. The skin was the most affected body region, with skin fold dermatitis, pyoderma and pododermatitis in the top ten most common disorders of French Bulldogs. These results provide a framework to identify health priorities in French Bulldogs and can contribute positively to reforms to improve health and welfare within the breed.

## Abbreviations

BOAS: Brachycephalic obstructive airway syndrome
CI: Confidence interval
EPR: Electronic patient record
IQR: Interquartile range
KC: The Kennel Club
OR: Odds ratio
URT: Upper respiratory tract
